# Genital HSV Shedding among Kenyan Women Initiating Antiretroviral Therapy

**DOI:** 10.1371/journal.pone.0163541

**Published:** 2016-09-28

**Authors:** Griffins O. Manguro, Linnet N. Masese, Ruth W. Deya, Amalia Magaret, Anna Wald, R. Scott McClelland, Susan M. Graham

**Affiliations:** 1 Kenyatta National Hospital, Nairobi, Kenya; 2 Department of Epidemiology, University of Washington, Seattle, Washington, United States of America; 3 Institute of Tropical and Infectious Diseases, University of Nairobi, Nairobi, Kenya; 4 Department of Laboratory Medicine, University of Washington, Seattle, Washington, United States of America; 5 Department of Medicine, University of Washington, Seattle, Washington, United States of America; 6 Fred Hutchinson Cancer Research Center, Seattle, Washington, United States of America; 7 Department of Global Health, University of Washington, Seattle, Washington, United States of America; David Geffen School of Medicine at UCLA, UNITED STATES

## Abstract

**Objectives:**

Genital ulcer disease (GUD) prevalence increases in the first month of antiretroviral treatment (ART), followed by a return to baseline prevalence by month 3. Since most GUD is caused by herpes simplex virus type 2 (HSV-2), we hypothesized that genital HSV detection would follow a similar pattern after treatment initiation.

**Methods:**

We conducted a prospective cohort study of 122 HSV-2 and HIV-1 co-infected women with advanced HIV disease who initiated ART and were followed closely with collection of genital swab specimens for the first three months of treatment.

**Results:**

At baseline, the HSV detection rate was 32%, without significant increase in genital HSV detection noted during the first month or the third month of ART. HIV-1 shedding declined during this period; no association was also noted between HSV and HIV-1 shedding during this period.

**Conclusion:**

Because other studies have reported increased HSV detection in women initiating ART and we have previously reported an increase in GUD during early ART, it may be prudent to counsel HIV-1 infected women initiating ART that HSV shedding in the genital tract may continue after ART initiation.

## Introduction

Herpes simplex virus (HSV) is a common sexually transmitted infection (STI) among HIV-1 infected persons [1, 2]. With widespread access to antiretroviral treatment (ART) and the success of this treatment in reducing genital HIV-1 shedding [3], attention has shifted to the impact of ART on other genital conditions, including genital ulcer diseases (GUD) and HSV shedding.

We previously reported a significant increase in GUD prevalence among HIV-1 infected women with advanced disease in the first month of ART, compatible with immune reconstitution inflammatory syndrome, followed by a return to baseline by the end of the third month [4]. Given that most cases of GUD in this population are likely caused by HSV [5], we hypothesized that HSV detection would follow a similar pattern after treatment initiation.

In this study, we analyzed prospectively collected data and tested genital swab samples for the presence of HSV DNA and HIV-1 RNA among women co-infected with HSV-2 and HIV-1. Our objective was to determine the pattern of genital HSV detection in co-infected women in the early months following ART initiation and its relationship to genital HIV-1 shedding.

## Materials and Methods

### Study design

We conducted a prospective study of HSV-2 seropositive women with advanced HIV disease who initiated ART. The women were part of an ongoing cohort study of female sex workers in Mombasa, Kenya.

### Study population and visit schedules

Data were collected from women who participated between March 2004 and September 2007 in a study to evaluate the effect of ART initiation on genital HIV-1 shedding. Women were invited to participate if they were eligible to initiate ART according to WHO and Kenyan national guidelines at that time (CD4 count <200 cells/μL or WHO stage 4 disease). All women initiated a standard treatment regimen of stavudine or zidovudine, lamivudine and nevirapine.

Data were collected from two different, non-overlapping populations of participating women. Population 1 comprised a subgroup of 21 women who consented to intensive follow-up in the first month of treatment. After screening and enrollment, follow-up visits with genital HSV swab collection were on days 1, 2, 4, 7, 14, and 28. Data from this population were used in the analysis of genital HSV detection in the first month of ART. Population 2 comprised 102 women who were followed monthly after ART initiation, with collection of swabs for genital HSV detection at baseline and month 3. Data from this population were used to evaluate changes in genital HSV detection and the association between genital HSV and HIV-1 detection at baseline and at month 3 of ART.

### Clinical procedures

Visit procedures were standard and identical for both populations. At enrollment and at each subsequent visit, participants underwent a questionnaire-guided, face-to-face interview followed by a standardized physical examination which included a pelvic speculum examination and screening for genital infections. Genital specimens for HSV detection were collected by inserting a Dacron swab into the cervical os, then rolling it firmly along the lateral fornix, vaginal wall, vulva, labial region, and finally the perianal region. Swab samples were placed in 1 mL of PCR digestion buffer and stored at -70°C until shipment to Seattle. Details on the specimen collection procedure for genital HIV-1 detection have been described elsewhere [3].

### Laboratory methods

HSV-2 serology testing was performed on archived frozen specimens using a type-specific, HSV-2 IgG-based ELISA (HerpeSelect, Focus Diagnostics, Cyprus, CA, USA). HSV-2 seropositivity was determined as an index value (the ratio of the optical density of the sample to that of a standard calibrator) >1.1, according to the manufacturer’s instructions [6]. At the University of Washington DNA was extracted from 200 μL of swab sample buffer with a QIAamp 96 DNA Blood Kit (Qiagen). Quantitative real-time PCR (QuantiTect multiplex PCR master mix from Qiagen) was performed with a 7900HT sequencing detection system using a validated assay with primers to the HSV gB region [7]. A positive assay was defined as detection of ≥150 copies of HSV DNA/mL of swab fluid [8]. HIV-1 RNA in genital secretions was quantified using the Hologic HIV-1 RNA assay (Hologic/Gen-Probe, San Diego, California, USA), with a lower limit of HIV-1 quantitation of 100 copies/swab. CD4 cell count was determined using Facscount (Becton Dickinson, Franklin Lakes, NJ, USA).

After Gram staining of cervical secretions, the number of polymorphonuclear leukocytes in three non-adjacent oil immersion fields on microscopy was quantified. Yeast and *T*. *vaginalis* were detected by light microscopy of vaginal saline wet preparations. BV was evaluated by Nugent scoring of a vaginal Gram stain.

### Statistical methods

The primary outcome was the frequency of genital HSV detection at the pre-ART baseline and at each time point after ART initiation. HIV-1 RNA was set at half the lower limit of detection (50 copies/swab) for samples with a result below 100 copies/swab. Because HIV-1 RNA values were not normally distributed, they were log_10_-transformed to approximate normality. Linear mixed effects models with a Poisson link were used to analyze associations with HSV detection in each study population, as such models provide estimates of within-person (marginal) effects following treatment initiation and produce results in the form of an incidence rate ratio, which is more easily interpreted than an odds ratio.

We selected two primary predictors to test: time point and genital HIV-1 shedding. For population 1, each participant contributed one row per time point assessed for HSV shedding (i.e., baseline and days 1, 2, 4, 7, 14 and 28). For population 2, participants contributed one row for baseline and one row for the month 3 visit. Time point was assessed using a series of indicator variables. Two different approaches were taken to analyze genital HIV-1 as a predictor of HSV detection. First, log_10_-tranformed vaginal viral load (VVL) and cervical viral load (CVL) were analyzed as separate predictors of genital HSV detection. Second, these two variables (i.e., vaginal and cervical HIV-1 viral load) were combined into one dichotomous variable (genital HIV-1 detected versus not detected), which was then analyzed for its association with genital HSV detection.

Potential confounders analyzed in bivariable analysis for their association with genital HSV detection at any time point included baseline CD4 count, WHO clinical stage at treatment initiation, hormonal contraception use, menstrual status, douching with soap, and the presence of vaginal infections or cervicitis. Final multivariable analyses were performed with time point, cervical and vaginal viral loads (model 1) or genital HIV-1 detection (model 2), adjusted for any potential confounder that was associated with HSV detection at a p value ≤0.10 in univariate analysis. Finally, Chi squared or Fisher’s exact tests were used to compare binary outcomes (genital HIV or HSV detection) at baseline and month 3.

### Ethics statement

The study was approved by the ethical review committees of the Kenya Medical Research Institute, the University of Washington, and the Fred Hutchinson Cancer Research Center. Prior to obtaining approval, all consent forms, case report forms and other data collection tools were reviewed by the IRBs. All participants gave written informed consent.

## Results

### Baseline characteristics and retention

Baseline demographic and clinical characteristics including median age, years of education, and CD4 count at ART initiation were broadly similar between the two populations (Table 1). Of 21 women enrolled in population 1, 20 (95.2%) completed one month follow-up with only one missed visit. One woman withdrew after visit 4 due to illness. Of 102 women enrolled in population 2, 96 (94.1%) remained on treatment at month 3. One woman in this population was HSV-2 seronegative at baseline and was excluded from analysis. All other women were HSV-2 seropositive with index values between 2 and 18.6 (all but 5 [4%] had values >3.5).

**Table 1:** Baseline Characteristics of HSV-2-Seropositive Women Initiating ART.

Characteristic	Population 1 N = 21 Median (IQR) or Number (percent)	Population 2 N = 101 Median (IQR) or Number (Percent)
**Demographics**		
Age (years)	35 (29–38)	36 (32–40)
Education (years)	8 (7–11)	7 (6–9)
Marital status		
Currently married	0	1 (1%)
Widowed/divorced/separated	12 (57%)	73 (74%)
Never married	9 (43%)	27 (26%)
**Contraceptive use**		
No contraceptive method	15 (71%)	65 (64%)
Hormonal contraceptive use		
OCP	0	5 (5%)
DMPA	6 (29%)	18 (18%)
Norplant	0	5 (5%)
Other contraceptives		
Condoms	0	3 (3%)
Bilateral tubal ligation	0	5 (5%)
Hysterectomy	0	1 (1%)
**Clinical characteristics**		
CD4 count (cells/μL)	121 (58–161)	122 (78–164)
Viral load (log_10_ copies/mL)		
Plasma viral load	5.34 (5.11–5.81)	5.55 (5.22–5.96)
Cervical viral load	4.00 (3.33–4.49)	4.04 (3.46–4.65)
Vaginal viral load	4.07 (3.69–4.66)	3.98 (3.03–4.57)
Genital HIV-1 RNA detection	21 (100%)	100 (99%)
WHO clinical staging		
Stage 1	2 (10%)	18 (18%)
Stage 2	4 (19%)	29 (29%)
Stage 3	13 (62%)	43 (43%)
Stage 4	2 (10%)	11 (11%)
Self-reported ulcer in past month	1 (5%)	5 (5%)
Genital ulcer on physical exam	1 (5%)	8 (8%)
Genital HSV detection	8 (38%)	32 (32%)

### Genital HSV detection in the first month of ART (population 1)

**Fig 1:**
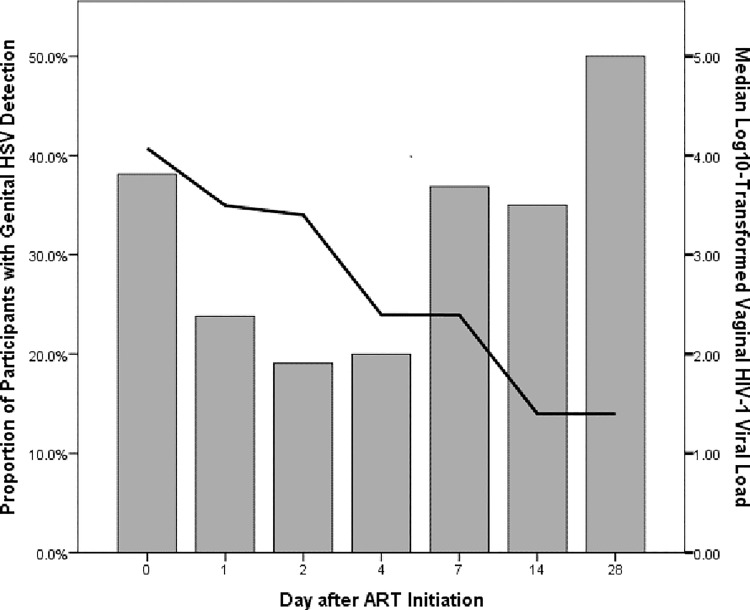
Genital HIV-1 shedding and HSV detection during month one of ART. Line graph depicting the median vaginal HIV-1 viral load superimposed on a bar graph depicting the proportion of participants in whom genital HSV was detected at each time point.

Genital HSV was detected in 8 of 21 (38.1%) women at baseline and in 10 of 20 (50%) women at day 28. Overall, genital HSV was detected at 45 of 142 (31.7%) visits in the first month of ART. At each visit, genital HSV detection ranged between 19% and 50%. When detected, HSV DNA levels ranged from 2.20 to 7.53 log_10_, with a median of 4.36 log_10_. One of two women with HerpeSelect index values <3.5 had genital HSV detected at two visits. HSV detection was somewhat less frequent on days 1–4 and somewhat more frequent at day 28 than at baseline (Fig 1).

Associations with HSV detection at any of the seven timepoints during the first month of ART are presented in Table 2. In bivariable analysis, there were no significant differences in genital HSV detection after ART initiation, compared to baseline. While the estimate of the incidence rate ratio was highest at day 28, this was not statistically significant (IRR 1.30, 95% CI 0.51–3.30). Genital HIV-1 shedding, whether analyzed as a continuous or binary variable, was not associated with genital HSV detection in the first month of ART (p = 0.53 for CVL, p = 0.78 for VVL and p = 0.27 for genital HIV-1). Of the potential confounders evaluated, only CD4 count of less than 100/mm^3^ at initiation of treatment was associated with increased genital HSV detection in the first month of ART (p = 0.01). In multivariable analysis with the *a priori* inclusion of log_10_ vaginal and cervical HIV-1 viral load and of baseline CD4 count and bacterial vaginosis based on p values, there was still no significant change from baseline in HSV detection at any time point during the first month of ART, including at day 28 (adjusted IRR 1.64, 95% CI 0.43–6.33).

**Table 2:** Characteristics Associated with Genital HSV Detection in Population 1.

Variable	Bivariable Analysis		Multivariable Analysis	
	Incidence Rate Ratio (95% CI)	p-value	Incidence Rate Ratio (95% CI)	p-value
Timepoint				
Baseline	Reference		Reference	
Day 1	0.62 (0.20–1.91)	0.41	0.71 (0.23–2.24)	0.56
Day 2	0.50 (0.15–1.66)	0.26	0.55 (0.15–1.96)	0.36
Day 4	0.52 (0.16–1.73)	0.29	0.61 (0.16–2.31)	0.47
Day 7	0.95 (0.35–2.63)	0.93	1.06 (0.30–3.79)	0.93
Day 14	0.91 (0.33–2.52)	0.86	1.14 (0.27–4.89)	0.86
Day 28	1.30 (0.51–3.30)	0.58	1.64 (0.43–6.33)	0.47
Log_10_-transformed cervical HIV-1 viral load	0.92 (0.71–1.20)	0.53	0.96 (0.60–1.54)	0.86
Log_10_-transformed vaginal HIV-1 viral load	0.96 (0.75–1.24)	0.78	1.13 (0.74–1.72)	0.57
Genital HIV-1 RNA detection	0.70 (0.37–1.32)	0.27		
Log_10_-transformed plasma HIV-1 viral load	0.91 (0.67–1.23)	0.53		
Clinical Stage				
Stage 1	Reference			
Stage 2	2.28 (0.48–10.74)	0.30		
Stage 3	1.19 (0.26–5.34)	0.82		
Stage 4	2.81 (0.55–14.28)	0.21		
Baseline CD4 count <100	2.32 (1.27–4.23)	0.01	2.28 (1.24–4.18)	0.008
Age	0.99 (0.93–1.05)	0.75		
Hormonal contraception use*	1.12 (0.53–2.39)	0.77		
Menstrual status				
1–3 weeks post-menses	Reference			
4–5 weeks post-menses	1.00 (0.34–2.92)	0.99		
Amenorrheic	0.79 (0.36–1.73)	0.56		
Douching with soap	0.83 (0.42–1.66)	0.61		
Bacterial vaginosis	0.57 (0.29–1.09)	0.09	0.63 (0.34–1.18)	0.15
Vulvovaginal candidiasis	0.66 (0.27–1.63)	0.37		

*Hormonal contraceptives included OCP, DMPA and Norplant

### Genital HSV detection at month 3 of ART (population 2)

Genital HSV was detected in 32 of 100 women (32.0%) at baseline, in 36 of 95 women (37.9%) at month 3, and overall during 68 of 195 (34.9%) of study visits by population 2. When detected, HSV DNA levels ranged from 2.27 to 7.62 log_10_, with a median of 4.69 log_10_. All three women with HerpeSelect index values <3.5 had genital HSV detected on at least one visit. Table 3 shows the analysis of associations with HSV detection at baseline and month 3 after ART initiation. In bivariable analysis, there was no difference in genital HSV detection at month 3 compared to baseline (IRR 1.18, 95% CI 0.74–1.91). Genital HIV-1 shedding, analyzed as either a continuous or a binary variable, was not associated with genital HSV detection at baseline or month 3 (p = 0.64 for CVL, p = 0.85 for VVL and p = 0.37 for genital HIV-1). In multivariable analysis with the *a priori* inclusion of log_10_ vaginal and cervical HIV-1 viral load and baseline CD4 count (based on results in Population 1), there was no significant change from baseline to month 3 in genital HSV detection (adjusted IRR 1.43, 95% CI 0.51–3.97).

**Table 3:** Associations with Genital HSV Detection in Population 2.

Variable	Bivariable Analysis		Multivariable Analysis	
	Incidence Rate Ratio (95% CI)	p-value	Incidence Rate Ratio (95% CI)	p-value
Timepoint				
Baseline	reference		reference	
Month 3	1.18 (0.74–1.91)	0.49	1.43 (0.51–3.97)	0.50
Log_10_-transformed cervical viral load	0.96 (0.80–1.15)	0.64	0.96 (0.68–1.36)	0.83
Log_10_-transformed vaginal viral load	1.02 (0.85–1.23)	0.85	1.19 (0.86–1.65)	0.50
Genital HIV-1 RNA detection	0.80 (0.48–1.31)	0.37		
Log_10_-transformed plasma viral load	0.97 (0.84–1.11)	0.62		
Clinical Stage				
Stage 1	reference			
Stage 2	1.32 (0.57–3.09)	0.52		
Stage 3	1.82 (0.85–3.93)	0.12		
Stage 4	1.59 (0.60–4.24)	0.35		
Baseline CD4 count <100	0.97 (0.59–1.59)	0.91	0.73 (0.38–1.41)	0.35
Age	0.98 (0.94–1.02)	0.32		
Hormonal contraception use*	1.41 (0.85–2.32)	0.18		
Menstrual status				
1–3 weeks post-menses	reference			
4–5 weeks post-menses	1.21 (0.65–2.28)	0.55		
Amenorrheic	0.93 (0.52–1.66)	0.80		
Douching with soap	1.04 (0.65–1.68)	0.86		
Bacterial vaginosis	1.00 (0.62–1.62)	0.99		
Vulvovaginal candidiasis	1.17 (0.63–2.19)	0.62		
Trichomoniasis	1.28 (0.47–3.53)	0.63		
Non-specific cervicitis	1.43 (0.20–10.27)	0.72		

*Hormonal contraceptives included OCP, DMPA and Norplant

### GUD, genital HSV and HIV-1 detection

At baseline, 100 women (99%) in population 2 had genital HIV-1 detected compared to 32 (32.0%) who had genital HSV detected. At month 3, genital HIV-1 detection reduced by more than half (37 women, 39.0%), in contrast to a slight increase in genital HSV detection (36 women, 37.9%). There was no association between genital HSV and HIV-1 detection at either day 0 or month 3 (p = 0.32 and p = 0.66, respectively). Baseline genital HSV detection had a borderline association with genital HSV detection at month 3 (p = 0.06), but no association with genital HIV-1 detection at month 3 (p = 0.69). A visible ulcer on physical examination was associated with genital HSV detection (p = 0.012), but not with genital HIV-1 detection (p = 0.76).

## Discussion

In this study, we describe genital HSV detection in HIV-1-and HSV-2-co-infected women at several time points during the first three months of ART, along with its association with genital HIV-1 shedding. Genital shedding of HSV was very frequent, occurring at up to 50% of visits at some time points and overall on 31.7 and 34.9% of visits for the two populations respectively. The prevalence of genital HSV detection decreased from baseline during the first 4 days of ART initiation, then was somewhat higher than baseline at day 28 and month 3, but these changes in prevalence did not reach statistical significance. Genital HIV-1 shedding at ART initiation was also not associated with genital HSV detection during this period. In general, genital HSV shedding persisted at levels similar to baseline in the setting of rapidly declining genital HIV-1 RNA levels.

Data on the effect of ART on genital HSV shedding are inconclusive. Four studies have reported a significant effect of ART in reducing clinical reactivations associated with HSV, but no significant effect on genital HSV shedding [9–12]. One cohort study reported a significant decrease in HSV-2 detection with ART use [13]. It is worth noting that these studies evaluated patients taking ART for several months or years. One recent study by Tobian et al, however, followed HIV-1-infected women prospectively from ART initiation and reported an increase in genital HSV-2 detection within the first three months after ART initiation (OR 1.94; 95% CI, 1.04–3.62) [14]. With the exception of our study, this is the only other study to date that has sought to address the question of changes in genital HSV detection following ART initiation and to evaluate the possibility of genital HSV shedding as a manifestation of ART-associated immune reconstitution inflammatory syndrome (IRIS) [14].

This latter study, by Tobian et al [14], shares a number of broad similarities to our study, notably the ART regimen (WHO first-line therapy in both studies), sample size (96 in Tobian et al vs. 100 in our study population 2) and median age of study participants (34.5 in Tobian et al vs. 36 in our study). A number of key differences, however, exist between the two studies which could potentially account for the difference in findings. First, the women in our study had more advanced immunosuppression (median CD4 cell count 136 vs. 217 in Tobian et al.), which may have influenced genital HSV shedding. It is plausible that with a lower mean lower CD4 cell count, the women in our cohort had higher pre-ART levels of genital HSV shedding, and therefore, any increase in genital HSV shedding observed following ART initiation would have been modest. Second, we report a higher level of genital HSV detection throughout the study (31.7% vs. 4.9% in Tobian et al in the first month and 34.5% vs. 14.9% in Tobian et al at month 3) [14]. The higher detection rate in our study could potentially be due to our more thorough sampling of genital areas, compared to the self-collected vaginal swab used by Tobian et al. Third, Tobian et al tested vaginal swabs for HSV-2 at several time points both before and after ART initiation, while we had stored genital swabs for this purpose only at baseline and month 3 for our larger study population (population 2). We therefore likely had less power to detect an increase in genital HSV-2 shedding after ART initiation than Tobian et al. did [14].

We found no association between genital HSV and genital HIV-1 detection at any time point, in contrast to studies of women not taking ART [15, 16]. In addition, baseline genital HSV detection was only weakly associated with genital HSV detection at month 3 of ART. That we found no increase in genital HSV detection among women with advanced HIV infection, in whom IRIS might be expected, is noteworthy; however, our statistical power was limited. In our prior research with this study population, we did find that genital ulceration on physical examination was strongly associated with HSV detection [4]. A recent paper by Fife et al has confirmed our finding of an increased risk of GUD in the first 3 months after ART initiation, and also verified that these ulcers are HSV-2-related [17].

Our study had several strengths. Participants were followed frequently in the first month in population 1, which enabled us to collect closely timed samples and detect changes in both genital HSV and genital HIV-1 from as early as the first day of treatment. Genital samples for HSV were also collected from an extensive area of the external genitalia, corresponding to where HSV-2 reactivates. Our study was limited by the small sample size of population 1. Because this study is exploratory, we did not correct for multiple comparisons. Finally, the study population included women with advanced immunosuppression, who may not be representative of the general population of women in Kenya or in other locations for this and other reasons.

In conclusion, we found that genital HSV detection was extremely prevalent both at baseline and at 3 months after ART initiation, without appreciable changes. Genital HSV detection was not associated with genital HIV-1 detection at any time point, nor did it predict future genital HIV-1 detection during the first three months of ART. Because other studies have reported increased HSV detection in women initiating ART and we and others have reported an increase in GUD during early ART, it may be prudent to counsel HIV-1 infected women initiating ART that HSV shedding in the genital tract may continue after ART initiation. However, our results suggest that HSV shedding does not substantially impact genital HIV-1 shedding in women who initiate ART.
